# Supplementary material for: Effect of forest cover change on the prevalence of acute respiratory tract infections, diarrhoea, and fever among children under five: Using an ecosystem approach to child health
Source: PLoS One. 2025 Aug 12;20(8):e0330101. doi: 10.1371/journal.pone.0330101 (PMC12342322; doi:10.1371/journal.pone.0330101)
Supplement: Attachment — Submitted filename: Comments.docx [file pone.0330101.s001.docx]

General comments:

This is an interesting study, providing a assessment of association between forest cover and infectious diseases in Nepal. Although the scale of the study is small, they provided a reasonable analysis and discussed their results well. I only have minor revision.

1. The line number should be provided in the article thoroughly.

2. The results in Table 3 should be provided as “estimate (95% CI)”.

3. What’s the criteria of covariates selection? It should be provided in method section.

4. Reference should be provided in the last paragraph in page 13.

5. Reference should be provided in the last paragraph in page 14.

6. For my consideration, it’s a cross-sectional study rather than ecological analysis.

7. I recommend authors to do stratify analysis according to different hygiene conditions, residential locations (urban/rural), and household income levels to identify effect modifiers and seek populations that could be more beneficial from forest cover.
