# Supplementary material for: Effect of forest cover change on the prevalence of acute respiratory tract infections, diarrhoea, and fever among children under five: Using an ecosystem approach to child health
Source: PLoS One. 2025 Aug 12;20(8):e0330101. doi: 10.1371/journal.pone.0330101 (PMC12342322; doi:10.1371/journal.pone.0330101)
Supplement: Attachment — Submitted filename: Response letter.docx [file pone.0330101.s002.docx]

22/12/2024

Dr Andrew G Wu

Academic Editor
PLOS ONE

**RE: Submission of the revised manuscript, PONE-D-24-38792**

Thank you for your time and feedback on our manuscript titled, ***Effect of Forest Cover Change on the Prevalence of Acute Respiratory Tract Infections, Diarrhoea, and Fever Among Children Under Five: Using an Ecosystem Approach to Child Health.***

We have carefully revised our manuscript and hope that the manuscript has improved substantially. Should you require any additional information, please do not hesitate to contact me.

Thank you for your time and consideration.

Sincerely,

Mr. Amit Timilsina, MPH

E-mail: timilsinaamit@gmail.com

**Reviewers' comments**

**Reviewer #1**

This is an interesting study, providing a assessment of association between forest cover and infectious diseases in Nepal. Although the scale of the study is small, they provided a reasonable analysis and discussed their results well. I only have minor revision.

1. The line number should be provided in the article thoroughly.

**RESPONSE: Amendment made.**

2. The results in Table 3 should be provided as “estimate (95% CI)”.

**RESPONSE: The format of Table 3 is revised to include results as “estimate (95% CI)”.**

3. What’s the criteria of covariates selection? It should be provided in method section.

**RESPONSE: Covariates included in the analysis were selected based on theoretical relevance, evidence from prior literature, and data availability. This information has been included in the methods section.**

4. Reference should be provided in the last paragraph in page 13.

**RESPONSE: References have been added.**

5. Reference should be provided in the last paragraph in page 14.

**RESPONSE: References have been added.**

6. For my consideration, it’s a cross-sectional study rather than ecological analysis.

**RESPONSE: Thank you, the amendment has been made.**

7. I recommend authors to do stratify analysis according to different hygiene conditions, residential locations (urban/rural), and household income levels to identify effect modifiers and seek populations that could be more beneficial from forest cover.

**RESPONSE: We appreciate the reviewer’s suggestion to conduct stratified analyses. However, we opted not to pursue these analyses as stratification would reduce statistical power due to smaller sample sizes within subgroups. We acknowledge the value of this approach and suggest it as a direction for future research.**

**Reviewer #2: Reviewer report PONE-D-24-38792**

This is a very interesting ecological study analysing the impact of forest canopy area on the prevalence of acute respiratory infections, fever and diarrhoea using country wide population health data (census) in Nepal.

The sampling framework enabled random samples of households to be recruited that are representative of the diverse topography and geography in this country.

I have a few questions for the authors to improve clarity and my understanding of their methods and findings.

Introduction

1. I would like to know more about the physiological or biological link between levels of forestation and its impact on childhood fever, ARI and diarrhoea so that the research question is more easily understood.

**RESPONSE: Thank you for your feedback. Explanations about the physiological and ecological mechanisms linking forest cover to childhood infections have been added to the Introduction section with references.**

Methods

2. Were all the covariates named in Table 1 used in the logit models as potential confounders?

**RESPONSE****: All covariates listed in Table 1 were included as potential confounders in the logit models. This has been mentioned in the statistical analysis section.**

3. Can the authors please explain how ethnicity was used as a measure of “advantage/disadvantage” (Page 8) – this might be obvious to the authors but I do not relate to ethnicity being analysed in this way.

**RESPONSE:** **The categorization of caste/ethnicity in DHS data for Nepal is based on the caste system, which includes more than 125 groups. Due to the socioeconomic status and cultural hierarchy of these castes and ethnicities in Nepalese society, the DHS classifies them into six groups: upper caste groups, relatively advantaged janajati, religious minorities, disadvantaged non-Dalit Terai castes, disadvantaged janajati, and Dalits. For the purpose of this study, we further divide these into two broad categories: advantaged ethnic groups (comprising upper caste groups and relatively advantaged janajati) and disadvantaged groups (encompassing all others).**

4. Why did the authors select a “logit” (logistic regression) model rather than a “generalized linear mixed model”

**RESPONSE:** **We used logistic regression with clustered standard errors at the village level because it is well-suited to the structure of our data, which comes from repeated cross-sectional surveys where households are not repeated across DHS waves. While households are nested within villages, the clustering in our data was appropriately accounted for by adjusting standard errors at the village (i.e. the DHS cluster) level, addressing intra-cluster correlation and ensuring valid inference. GLMMs are typically advantageous when analyzing hierarchical data with repeated measures or when random effects can capture substantial within-cluster variability. However, in this case, the absence of repeated measures for households and the cross-sectional nature of the data makes random effects unnecessary. Logistic regression with clustered standard errors provides a parsimonious and interpretable approach, aligning with our objective to assess population-level relationships while appropriately accounting for the data structure.**

5. Table 2: Only mean and standard error for each variable are reported – it is hard to understand the distribution of the variables. I would like to see more detailed summary statistics.

**RESPONSE: Thank you. We acknowledge the value of additional statistics however, we opted to present the means and standard errors as they align with the primary objectives of our analysis, which focus on comparative trends over time rather than detailed distributional characteristics, which are in fact available in Demographic Survey reports.**

Table 3:

6. The lay out of Table 3 is difficult to follow – what are the numbers in the brackets for the “Forest Cover” row? Are these p-values?

**RESPONSE: Table 3 has been revised for clarity.**

7. The ** p<0.01 – is this correctly reported in Table 3, if the numbers in the brackets are p-values? Please clarify this.

**RESPONSE: Amendment made.**

8. Why not report the 95%CI in the Table 3 – it would be helpful see the range?

**RESPONSE: The 95% confidence intervals of the predicted probabilities are added to Table 3 for all estimates.**

9. Why are there rows for covariates and what does “yes” mean?

**RESPONSE: Clarified in Table 3.**

10. The probability of lower levels of reported fever in 2016 is remarkably larger than in 2011 – am I reading the results correctly?

**RESPONSE: Yes you are correct.**

11. How was the change in probability from 2011 to 2016 calculated? I can see the formula but cannot see how the calculation was conducted.

**RESPONSE: The differences in average infection probabilities between the years 2011 and 2016 were calculated by including a dummy variable for the year 2016 in the regression model. This dummy variable takes the value of "1" for observations from the 2016 DHS wave and "0" for the 2011 wave. The coefficient of this dummy variable captures the change in the average probability of infection in 2016 relative to 2011, holding all other covariates constant. Specifically, the regression equation in page 7 can be expressed as:**

$$\boldsymbol{P}\left( \mathbf{infection}\mathbf{=1} | \mathbf{X} \right)\boldsymbol{= \Phi(}\boldsymbol{\beta}_{\mathbf{0}}\mathbf{+}\boldsymbol{\beta}_{\mathbf{1}}\mathbf{fcover+}\boldsymbol{\beta}_{\mathbf{2}}\mathbf{DHS2016}\mathbf{+}\boldsymbol{\ldots}\mathbf{)}$$

**Here,** $\boldsymbol{\beta}_{\mathbf{2}}\mathbf{DHS2016}$ **quantifies the difference in the baseline log-odds of infection between 2016 and 2011, controlling for forest cover, household socio-demographic characteristics, maternal and child health characteristics, and household environmental characteristics. The corresponding probability difference can be computed by transforming the predicted log-odds into probabilities using the inverse of the link function (in this case, the cumulative normal distribution, Φ).**

Minor issues

12. There are numerous grammatical and spelling errors throughout the manuscript, although they do not detract from the meaning of the manuscript, they will need to be addressed to improve readability.

**RESPONSE: The manuscript has undergone a thorough proofreading process to address grammatical and spelling errors.**
