# Supplementary material for: Effect of forest cover change on the prevalence of acute respiratory tract infections, diarrhoea, and fever among children under five: Using an ecosystem approach to child health
Source: PLoS One. 2025 Aug 12;20(8):e0330101. doi: 10.1371/journal.pone.0330101 (PMC12342322; doi:10.1371/journal.pone.0330101)
Supplement: Attachment — Submitted filename: Response_letter_auresp_2.docx [file pone.0330101.s003.docx]

13/03/2025

Dr Furqan Kabir

Academic Editor
PLOS ONE

**RE: Submission of the revision 2 of the manuscript, PONE-D-24-38792**

Thank you for your time and feedback on our manuscript titled, ***Effect of Forest Cover Change on the Prevalence of Acute Respiratory Tract Infections, Diarrhoea, and Fever Among Children Under Five: Using an Ecosystem Approach to Child Health.***

We have carefully revised our manuscript based on the feedback. Should you require any additional information, please do not hesitate to contact me.

Thank you for your time and consideration.

Sincerely,

Mr. Amit Timilsina, MPH

E-mail: timilsinaamit@gmail.com

**Reviewers' comments**

**Reviewer #2**

I thank the authors for responding to the reviewers’ comments and questions. Aside from some minor grammar edits, I recommend this manuscript be accepted for publication. I look forward to future research which tracks the trends identified in these analyses.

Grammar/edits

Line 95: ‘sub-region’ should be ‘sub-regions’

Line 96: ‘province’ should be ‘provinces’

Line 141: please add a comma after ‘dwelling’

Line 111: ArcGIS Pro requires some reference with the version number and manufacturer.

Line 180: Please consider adding “decreased” after “(5% to 3%) as the sentence is incomplete.

Line 180: Please edit the last sentence to “For detailed information, please refer to Table 2”.

**RESPONSE: Thank you. All the suggested amendments have been made.**

**Reviewer #3**

Thank you for the revised manuscript. Most of the comments from the first review were addressed. however- I still feel the following points still need to be addressed.

Page 3 line 50 – Are these global statistics? Please specify

**RESPONSE: Amendment made.**

PAGE 8 LINES 178- 180 The second part of the sentence seems incomplete

**RESPONSE: Amendment made.**

Page 11 lines 244-245 While I understand this reasoning, I do not think that forest cover is directly related to diarrhoea either.

**RESPONSE: Thank you. The paragraph has been revised for clarity.**

One of the challenges in understanding the results is the lack of sample statistics (n +%). This helps us understand the prevalence of the different conditions and could also help us understand the non-significant results. Although the authors provide reasons for presenting means and standard errors only in their response- including sample distribution could still enhance the interpretation of the results.

**RESPONSE: Thank you for your comment. We truly appreciate your time and feedback. However, we prefer to maintain a focused presentation in this paper on the primary trends and the implications of forest cover changes on childhood infections. As you know, there have been published numerous papers over the past two decades on childhood fever, ARI, and diarrhoea using DHS data, focusing on the detailed demographic and distributional context. We hope you will agree with our rationale and decision. Thank you once again.**
