# Supplementary material for: Effect of forest cover change on the prevalence of acute respiratory tract infections, diarrhoea, and fever among children under five: Using an ecosystem approach to child health
Source: PLoS One. 2025 Aug 12;20(8):e0330101. doi: 10.1371/journal.pone.0330101 (PMC12342322; doi:10.1371/journal.pone.0330101)
Supplement: Attachment — Submitted filename: Response letter_R4.docx [file pone.0330101.s004.docx]

11/04/2025

Dr Furqan Kabir

Academic Editor
PLOS ONE

**RE: Submission of the revision 3 of the manuscript, PONE-D-24-38792**

Thank you for your feedback on our manuscript titled, ***Effect of Forest Cover Change on the Prevalence of Acute Respiratory Tract Infections, Diarrhoea, and Fever Among Children Under Five: Using an Ecosystem Approach to Child Health.***  We have revised the reference list based on your comment.

Thank you for your time and consideration.

Sincerely,

Mr. Amit Timilsina, MPH

E-mail: timilsinaamit@gmail.com

**Journal Requirements:**

1. Please include your full ethics statement in the ‘Methods’ section of your manuscript file. In your statement, please include the full name of the IRB or ethics committee who approved or waived your study, as well as whether or not you obtained informed written or verbal consent. If consent was waived for your study, please include this information in your statement as well.

**RESPONSE: Thank you. Ethics statement included.**

2. Please review your reference list to ensure that it is complete and correct. If you have cited papers that have been retracted, please include the rationale for doing so in the manuscript text, or remove these references and replace them with relevant current references. Any changes to the reference list should be mentioned in the rebuttal letter that accompanies your revised manuscript. If you need to cite a retracted article, indicate the article’s retracted status in the References list and also include a citation and full reference for the retraction notice.

**RESPONSE: Thank you. We have carefully checked all the references. References 1, 2, 4, 17, 23, and 24 have been corrected.**
