# Supplementary material for: Effect of forest cover change on the prevalence of acute respiratory tract infections, diarrhoea, and fever among children under five: Using an ecosystem approach to child health
Source: PLoS One. 2025 Aug 12;20(8):e0330101. doi: 10.1371/journal.pone.0330101 (PMC12342322; doi:10.1371/journal.pone.0330101)
Supplement: Attachment — Submitted filename: Response letter_R6.docx [file pone.0330101.s005.docx]

24/04/2025

Maidelyn R. Peregrin

PLOS ONE

**RE: Submission of the revision 6 of the manuscript, PONE-D-24-38792**

Thank you for your feedback regarding the use of figures containing map-based data. After careful consideration, we have decided to remove Figures 1 and 2 from the manuscript.

Thank you for your time and consideration.

Sincerely,

Mr. Amit Timilsina, MPH

E-mail: timilsinaamit@gmail.com

**Journal Requirements:**

1. 1.      We note that Figures 1,2 and 3 in your submission contain [map/satellite] images which may be copyrighted. All PLOS content is published under the Creative Commons Attribution License (CC BY 4.0), which means that the manuscript, images, and Supporting Information files will be freely available online, and any third party is permitted to access, download, copy, distribute, and use these materials in any way, even commercially, with proper attribution. For these reasons, we cannot publish previously copyrighted maps or satellite images created using proprietary data, such as Google software (Google Maps, Street View, and Earth). For more information, see our copyright guidelines: [http://journals.plos.org/plosone/s/licenses-and-copyright](https://aus01.safelinks.protection.outlook.com/?url=http%3A%2F%2Fjournals.plos.org%2Fplosone%2Fs%2Flicenses-and-copyright&data=05%7C02%7Csuthapa%40csu.edu.au%7C4320f31b28974e8326e108dd81fe4043%7Cf0f76207a6104fc0b4a35d797fe5283c%7C0%7C0%7C638809651499959940%7CUnknown%7CTWFpbGZsb3d8eyJFbXB0eU1hcGkiOnRydWUsIlYiOiIwLjAuMDAwMCIsIlAiOiJXaW4zMiIsIkFOIjoiTWFpbCIsIldUIjoyfQ%3D%3D%7C0%7C%7C%7C&sdata=bbF4llFGZkVMEjqLU1yp5bmcLcG9Hyjc7m0tbiB8XUs%3D&reserved=0).

We require you to either (1) present written permission from the copyright holder to publish these figures specifically under the CC BY 4.0 license, or (2) remove the figures from your submission:

1. You may seek permission from the original copyright holder of Figures 1,2 and 3 to publish the content specifically under the CC BY 4.0 license. 

We recommend that you contact the original copyright holder with the Content Permission Form ([http://journals.plos.org/plosone/s/file?id=7c09/content-permission-form.pdf](https://aus01.safelinks.protection.outlook.com/?url=http%3A%2F%2Fjournals.plos.org%2Fplosone%2Fs%2Ffile%3Fid%3D7c09%2Fcontent-permission-form.pdf&data=05%7C02%7Csuthapa%40csu.edu.au%7C4320f31b28974e8326e108dd81fe4043%7Cf0f76207a6104fc0b4a35d797fe5283c%7C0%7C0%7C638809651499976287%7CUnknown%7CTWFpbGZsb3d8eyJFbXB0eU1hcGkiOnRydWUsIlYiOiIwLjAuMDAwMCIsIlAiOiJXaW4zMiIsIkFOIjoiTWFpbCIsIldUIjoyfQ%3D%3D%7C0%7C%7C%7C&sdata=gfYUHIRwGNyshX%2BD5xHK%2BjdoUYSqsNfq5khZJvNTfIg%3D&reserved=0)) and the following text:
“I request permission for the open-access journal PLOS ONE to publish XXX under the Creative Commons Attribution License (CCAL) CC BY 4.0 ([http://creativecommons.org/licenses/by/4.0/](https://aus01.safelinks.protection.outlook.com/?url=http%3A%2F%2Fcreativecommons.org%2Flicenses%2Fby%2F4.0%2F&data=05%7C02%7Csuthapa%40csu.edu.au%7C4320f31b28974e8326e108dd81fe4043%7Cf0f76207a6104fc0b4a35d797fe5283c%7C0%7C0%7C638809651499988648%7CUnknown%7CTWFpbGZsb3d8eyJFbXB0eU1hcGkiOnRydWUsIlYiOiIwLjAuMDAwMCIsIlAiOiJXaW4zMiIsIkFOIjoiTWFpbCIsIldUIjoyfQ%3D%3D%7C0%7C%7C%7C&sdata=Skg6dBKeTz0BLiEoTy9Nf2VucPyAX2k2lozLpUHy5fY%3D&reserved=0)). Please be aware that this license allows unrestricted use and distribution, even commercially, by third parties. Please reply and provide explicit written permission to publish XXX under a CC BY license and complete the attached form.”

Please upload the completed Content Permission Form or other proof of granted permissions as an ""Other"" file with your submission.

In the figure caption of the copyrighted figure, please include the following text: “Reprinted from [ref] under a CC BY license, with permission from [name of publisher], original copyright [original copyright year].”

2. If you are unable to obtain permission from the original copyright holder to publish these figures under the CC BY 4.0 license or if the copyright holder’s requirements are incompatible with the CC BY 4.0 license, please either i) remove the figure or ii) supply a replacement figure that complies with the CC BY 4.0 license. Please check copyright information on all replacement figures and update the figure caption with source information. If applicable, please specify in the figure caption text when a figure is similar but not identical to the original image and is therefore for illustrative purposes only.
The following resources for replacing copyrighted map figures may be helpful:

USGS National Map Viewer (public domain): [http://viewer.nationalmap.gov/viewer/](https://aus01.safelinks.protection.outlook.com/?url=http%3A%2F%2Fviewer.nationalmap.gov%2Fviewer%2F&data=05%7C02%7Csuthapa%40csu.edu.au%7C4320f31b28974e8326e108dd81fe4043%7Cf0f76207a6104fc0b4a35d797fe5283c%7C0%7C0%7C638809651500002855%7CUnknown%7CTWFpbGZsb3d8eyJFbXB0eU1hcGkiOnRydWUsIlYiOiIwLjAuMDAwMCIsIlAiOiJXaW4zMiIsIkFOIjoiTWFpbCIsIldUIjoyfQ%3D%3D%7C0%7C%7C%7C&sdata=cJ2ggpbkwhwT06rKMBZcg%2FZ85Fpt7StETqo5t1wXBBM%3D&reserved=0)
The Gateway to Astronaut Photography of Earth (public domain): [http://eol.jsc.nasa.gov/sseop/clickmap/](https://aus01.safelinks.protection.outlook.com/?url=http%3A%2F%2Feol.jsc.nasa.gov%2Fsseop%2Fclickmap%2F&data=05%7C02%7Csuthapa%40csu.edu.au%7C4320f31b28974e8326e108dd81fe4043%7Cf0f76207a6104fc0b4a35d797fe5283c%7C0%7C0%7C638809651500015083%7CUnknown%7CTWFpbGZsb3d8eyJFbXB0eU1hcGkiOnRydWUsIlYiOiIwLjAuMDAwMCIsIlAiOiJXaW4zMiIsIkFOIjoiTWFpbCIsIldUIjoyfQ%3D%3D%7C0%7C%7C%7C&sdata=j%2B2L4BChY6R4pjcU0GX3d0NQ9MABFqhHCOglaW153%2FU%3D&reserved=0)
Maps at the CIA (public domain): [https://www.cia.gov/library/publications/the-world-factbook/index.html](https://aus01.safelinks.protection.outlook.com/?url=https%3A%2F%2Fwww.cia.gov%2Flibrary%2Fpublications%2Fthe-world-factbook%2Findex.html&data=05%7C02%7Csuthapa%40csu.edu.au%7C4320f31b28974e8326e108dd81fe4043%7Cf0f76207a6104fc0b4a35d797fe5283c%7C0%7C0%7C638809651500027176%7CUnknown%7CTWFpbGZsb3d8eyJFbXB0eU1hcGkiOnRydWUsIlYiOiIwLjAuMDAwMCIsIlAiOiJXaW4zMiIsIkFOIjoiTWFpbCIsIldUIjoyfQ%3D%3D%7C0%7C%7C%7C&sdata=kyp51P5PMqa0aIRI1JU8lM%2BMbkX5XK0EzpJneHcPxBI%3D&reserved=0) and [https://www.cia.gov/library/publications/cia-maps-publications/index.html](https://aus01.safelinks.protection.outlook.com/?url=https%3A%2F%2Fwww.cia.gov%2Flibrary%2Fpublications%2Fcia-maps-publications%2Findex.html&data=05%7C02%7Csuthapa%40csu.edu.au%7C4320f31b28974e8326e108dd81fe4043%7Cf0f76207a6104fc0b4a35d797fe5283c%7C0%7C0%7C638809651500039647%7CUnknown%7CTWFpbGZsb3d8eyJFbXB0eU1hcGkiOnRydWUsIlYiOiIwLjAuMDAwMCIsIlAiOiJXaW4zMiIsIkFOIjoiTWFpbCIsIldUIjoyfQ%3D%3D%7C0%7C%7C%7C&sdata=i2e431cRMcAXBkiNL%2Bm5R1vzHY6v%2BmHOR5jKHx9usF0%3D&reserved=0)
NASA Earth Observatory (public domain): [http://earthobservatory.nasa.gov/](https://aus01.safelinks.protection.outlook.com/?url=http%3A%2F%2Fearthobservatory.nasa.gov%2F&data=05%7C02%7Csuthapa%40csu.edu.au%7C4320f31b28974e8326e108dd81fe4043%7Cf0f76207a6104fc0b4a35d797fe5283c%7C0%7C0%7C638809651500051577%7CUnknown%7CTWFpbGZsb3d8eyJFbXB0eU1hcGkiOnRydWUsIlYiOiIwLjAuMDAwMCIsIlAiOiJXaW4zMiIsIkFOIjoiTWFpbCIsIldUIjoyfQ%3D%3D%7C0%7C%7C%7C&sdata=iOetlh1PPZr2uqx%2FC5QArMB0ZYKG9FG%2BY1%2FxY2sg5%2FQ%3D&reserved=0)
Landsat: [http://landsat.visibleearth.nasa.gov/](https://aus01.safelinks.protection.outlook.com/?url=http%3A%2F%2Flandsat.visibleearth.nasa.gov%2F&data=05%7C02%7Csuthapa%40csu.edu.au%7C4320f31b28974e8326e108dd81fe4043%7Cf0f76207a6104fc0b4a35d797fe5283c%7C0%7C0%7C638809651500063649%7CUnknown%7CTWFpbGZsb3d8eyJFbXB0eU1hcGkiOnRydWUsIlYiOiIwLjAuMDAwMCIsIlAiOiJXaW4zMiIsIkFOIjoiTWFpbCIsIldUIjoyfQ%3D%3D%7C0%7C%7C%7C&sdata=fk528hN9RDYb0qUFGBN3355ohe2VSMqfSCShHao79EA%3D&reserved=0)
USGS EROS (Earth Resources Observatory and Science (EROS) Center) (public domain): [http://eros.usgs.gov/#](https://aus01.safelinks.protection.outlook.com/?url=http%3A%2F%2Feros.usgs.gov%2F%23&data=05%7C02%7Csuthapa%40csu.edu.au%7C4320f31b28974e8326e108dd81fe4043%7Cf0f76207a6104fc0b4a35d797fe5283c%7C0%7C0%7C638809651500075531%7CUnknown%7CTWFpbGZsb3d8eyJFbXB0eU1hcGkiOnRydWUsIlYiOiIwLjAuMDAwMCIsIlAiOiJXaW4zMiIsIkFOIjoiTWFpbCIsIldUIjoyfQ%3D%3D%7C0%7C%7C%7C&sdata=NXuSZuyvm8622FYARsJzLZq%2BI4JomwAtbMhJZyGe9Bk%3D&reserved=0)
Natural Earth (public domain): [http://www.naturalearthdata.com/](https://aus01.safelinks.protection.outlook.com/?url=http%3A%2F%2Fwww.naturalearthdata.com%2F&data=05%7C02%7Csuthapa%40csu.edu.au%7C4320f31b28974e8326e108dd81fe4043%7Cf0f76207a6104fc0b4a35d797fe5283c%7C0%7C0%7C638809651500087395%7CUnknown%7CTWFpbGZsb3d8eyJFbXB0eU1hcGkiOnRydWUsIlYiOiIwLjAuMDAwMCIsIlAiOiJXaW4zMiIsIkFOIjoiTWFpbCIsIldUIjoyfQ%3D%3D%7C0%7C%7C%7C&sdata=OaA02l%2BK7KQYkDllGsNkXf%2BIBIuDyPqUF%2FUdG5oGWu0%3D&reserved=0)"

**RESPONSE:** Thank you for your feedback regarding the use of figures containing map-based data. After careful consideration, we have decided to remove Figures 1 and 2 from the manuscript.

Our study primarily aims to examine the relationship between forest cover change and the risk of common childhood infections among children under five in Nepal. While spatial representation of forest cover was initially included for context, it is not central to our research objectives or analyses. We believe that removing these figures will not affect the clarity or scientific contribution of the study.

We appreciate your guidance and hope this revised version aligns better with the journal’s licensing requirements.
